# Antidiphtheritic Serum

**Published:** 1907-06-29

**Authors:** 


### Points in Treatment.

### ANTIDIPHTHERITIC SERUM.

There are three main objections to the use of antidiphtheritic serum in the treatment of diphtheria, namely:—

1. The cost of the serum.
2. The disinclination of the patient, or of the patient's friends, to hypodermic medication.
3. The possibility of a serum rash a week or ten days subsequent to the injection.

On the other hand, the benefit of the treatment is so certain that the above objections are quite minor in comparison. The cost of the serum is a few shillings, but when it is a question of purchasing the life of a child for these few shillings, as may be the case, there are few who cannot afford them. The disinclination to hypodermic medication is natural, and if there were any other way of getting equally good results from the serum its administration hypodermically would not be resorted to. Attempts have been made to use it per rectum, but there is no real proof that it is efficacious when given so, whereas there are ample statistics to show that hypodermic administration cures; when front to front with diphtheria, therefore, it is only when there is a point-blank and invincible refusal to have the serum that it should not be given hypodermically. Should a serum rash follow in any particular patient it is the duty of the medical man to notify the fact to the manufacturers of the serum, giving the number of the phial; the manufacturers can then trace the serum to its source, and ascertain which horse it is whose blood contains the irritant material; they can then stop preparing serum from that particular horse, for there are other horses whose serum seldom or never gives rise to serum rashes, and it is from these that the antitoxin should be prepared.

### The Mode in which Diphtheria Toxin Acts.

It is often held that the antitoxin need only be used in the cases in which the diphtheria itself is severe. It is urged, for example: "We did not give the serum here because the symptoms of the diphtheria were slight; it seemed to be an ordinary simple case, and we did not feel justified in putting the parents to the expense of serum treatment." It is true that the great majority of diphtheria cases will get well without serum treatment. This, however, is not the highest point of view. The question is: "Will my patient be more likely to survive if serum is given than if it is not?" The answer is: "Provided the serum is given *early*, the chances of my patient's survival are greatly increased; if I give the serum at the very beginning it is almost certain that my patient will not die of this diphtheria." Great emphasis must be laid upon the word *early* here, and for this reason:—

Diphtheria toxin is produced by the bacilli at the place where the latter are growing—the fauces, the nose, the larynx, the conjunctiva, and so on. When first produced, the toxin is free; it enters the blood-stream free, and gets carried in the circulation to all parts of the body. *It does not remain free long.* When it comes in contact with the susceptible body-cells it becomes *chemically fixed* to the latter; the toxin no longer exists as such, but the cells to which it is combined are crippled and damaged. As time goes on, more and more toxin is produced, more and more cells are damaged by the attachment of toxin to them. The patient's body as a whole can go on living in spite of the combination of toxin with cells up to a certain point, but there is a limit beyond which continuation of life is impossible. The fact that an attack of diphtheria appears to be mild when it is first seen is no guarantee that the production of toxin by the bacteria in that particular patient will cease before this limit is reached. To wait until the symptoms, by their severity, indicate that the limit is being approached, is to wait until the house has nearly burned to the ground before trying to put out the fire.

### The Mode in which Diphtheria Antitoxin Acts.

Diphtheria antitoxin acts as a true antidote to the diphtheria toxin; that is to say, the antitoxin combines with the toxin, the compound of the two being harmless. The antitoxin, however, can only combine with the toxin when the latter is *free*; as soon as the toxin has become combined with the protoplasm of the body cells the antitoxin is unable to touch it; the harm is done, and the antitoxin is helpless in remedying it. Once a fatal dose of toxin has been formed, and has united with the living cells of the body, no treatment can save the patient. The antitoxin, to be of use, must be given before the combination of toxin with tissue cells has taken place; it must therefore be given early; it has been found at fever hospitals that, when antidiphtheritic serum is given on the first day of the diphtheria, not a single patient dies; when the opportunity for giving the serum does not occur until the second day of the disease there is a small mortality, but much less than if serum treatment is not adopted; if it is the third day before serum can be given, the mortality is only slightly less than it is when serum is not given; from the fourth day onwards the mortality is just about the same, whether serum is given or not. The reason for this is clear: During the four days the bacilli have been producing toxin which has been carried to the tissue cells by the blood, and which has there combined with those cells, the combination is strong and cannot be undone by the antitoxin. It is our duty to give the antitoxin, wherever possible, as near to the beginning of the illness as may be; to give it without waiting for the result of any bacteriological confirmation of the diagnosis to give it in sufficient quantity not only to neutralise any toxin that may actually be present free at the time, but also to be there ready to combine with the toxin that is going to be produced during the succeeding hours and days.

